# Omega‐3 fatty acid supplementation does not attenuate declines in skeletal muscle mitochondrial area in young, healthy females during immobilization

**DOI:** 10.14814/phy2.70736

**Published:** 2026-01-23

**Authors:** Megan M. Lo, Merryl N. Black, Chris McGlory, Ravninder Bahniwal, Michael Kamal, Joe Quadrilatero, Stuart M. Phillips, Michaela C. Devries

**Affiliations:** ^1^ Department of Kinesiology and Health Sciences University of Waterloo Waterloo Canada; ^2^ Department of Kinesiology McMaster University Hamilton Canada

**Keywords:** electron microscopy, mitochondria, muscle disuse, omega‐3 fatty acid, skeletal muscle

## Abstract

Mitochondrial subcellular area influences function. Muscle disuse reduces mitochondrial content; however, its effect on mitochondrial subcellular location is unclear. Omega‐3 fatty acid (n‐3) attenuates declines in muscle mass and mitochondrial function during disuse; however, whether n‐3 supplementation prevents the decline in mitochondrial content has not been examined. We investigated the effects of 2 weeks of leg immobilization followed by 2 weeks of remobilization on skeletal muscle mitochondrial content and subcellular localization with and without n‐3 supplementation. Twenty healthy females supplemented with n‐3 (2.97 g EPA and 2.03 g DHA) or control (isoenergetic sunflower oil) during 2 weeks of unilateral leg immobilization and 2 weeks of remobilization. *Vastus lateralis* biopsies were taken for electron microscopic analysis of mitochondrial content. Subsarcolemmal (SS) mitochondrial content decreased during immobilization (control: −9%, n‐3: −66%, *p* = 0.009) and remained lower following recovery (control: −41%, n‐3: −42%, *p* = 0.005). This effect was driven by the n‐3 group (*p* < 0.02). Intermyofibrillar (IMF) mitochondrial content did not decline during immobilization, but was lower than baseline following recovery in the central (*p* = 0.01) IMF. The effects of leg immobilization on mitochondrial content differ by location, are not reversed with short‐term recovery, and are influenced by n‐3 supplementation.

## 1 INTRODUCTION

Periods of muscle disuse occur throughout the lifespan due to illness and injury leading to a loss of muscle mass and strength (Wall et al., 2013). The mechanisms that underpin disuse‐induced muscle atrophy are multifactorial and include declines in muscle protein synthesis (Deane et al., 2024; Kilroe et al., 2020; Tesch et al., 2008), increases in proteolysis (Tesch et al., 2008), and reductions in mitochondrial content and function (Hyatt et al., 2019). Specifically, declines in mitochondrial content and enzyme activity have been observed in rodent models of disuse, including hind limb suspension and denervation (Hood et al., 2019; Memme et al., 2021; Tryon et al., 2014). Moreover, 2 weeks of unilateral limb immobilization has been reported to reduce mitochondrial respiratory capacity (Gram et al., 2014; Miotto et al., 2019) in both young and older human males, as well as in young females in a cohort paper to this study. Moreover, the recovery of mitochondrial function appears to be impaired upon remobilization, as there is an incomplete recovery of mitochondrial‐related genes following recovery from 2‐weeks of reduced step count in older human adults (McGlory et al., 2018). Furthermore, while other markers of mitochondrial biogenesis, fission and autophagy recover with reambulation, BNIP3L, a mitophagy protein involved in the clearance of damaged mitochondria, expression fails to normalize in recovery after 2‐weeks of unilateral limb immobilization in middle‐aged males (Pileggi et al., 2023). Mitochondria are dynamic organelles, playing key roles in the production of energy and maintaining cellular homeostasis (Qualls et al., 2021), thus identifying strategies to attenuate declines in mitochondrial content and function induced by disuse and promote recovery are critical.

Within skeletal muscle, mitochondria are found in the subsarcolemmal (SS) and intermyofibrillar (IMF) regions (Cogswell et al., 1993; Ferreira et al., 2010). While there is an effect of exercise training to increase mitochondrial content in both the IMF and SS regions, the effect of training on mitochondrial content is more pronounced in the SS region (Howald et al., 1985; Nielsen, Mogensen, et al., 2010). Although mitochondrial content declines during disuse, it remains unclear whether this is due to a greater effect on SS or IMF mitochondria. One trial reported a decline in IMF, but not SS mitochondria, following 42 days of bed rest in young healthy males (Ferretti et al., 1997), while another study reported a decline in SS, but not IMF mitochondria, following 2 weeks of single‐leg immobilization in young and older males (Nielsen, Suetta, et al., 2010). However, no study has investigated how disuse influences mitochondrial subcellular localization in females or how subcellular mitochondrial localization recovers following disuse. Given that previous studies have reported that subcellular localization influences mitochondrial function (Ferreira et al., 2010; Ritov et al., 2005), understanding how disuse affects mitochondrial localization is crucial for comprehending the overall pathology of disuse and for developing strategies to mitigate these effects.

Long chain omega‐3 (n‐3) fatty acid (FA) supplementation improves mitochondrial ADP sensitivity by altering membrane lipid composition (Herbst et al., 2014) in healthy males. In the same cohort as our present study, n‐3 supplementation attenuated skeletal muscle atrophy and facilitated muscle mass recovery during remobilization, which was linked to the partial preservation of myofibrillar protein synthesis (Mcglory et al., 2019). Furthermore, in this same cohort, n‐3 supplementation prevented reductions in mitochondrial coupled respiration and electron transport chain proteins following 14 days of immobilization (Miotto et al., 2019). Thus, n‐3 supplementation may represent an effective strategy to mitigate immobilization‐related declines in mitochondrial health. Given the differential responsiveness of mitochondria in distinct subcellular compartments to stimuli such as exercise (Howald et al., 1985; Nielsen, Mogensen, et al., 2010), there may be unique responses to disuse, recovery, and supplementation from different subcellular mitochondrial populations; however, this remains to be examined.

We aimed to characterize the effects of 2‐weeks of immobilization followed by 2‐weeks of remobilization on mitochondrial subcellular localization and content in young, healthy females and determine whether any deleterious effects of disuse were offset by n‐3 supplementation. We hypothesized that disuse would induce a decline in SS, but not IMF mitochondrial content and that SS mitochondrial content would remain below baseline following 2‐weeks of remobilization. We hypothesized that n‐3 supplementation during immobilization would attenuate the declines in SS mitochondrial content, allowing mitochondrial content to return to baseline by the end of the remobilization period.

## 2 METHODS

### 2.1 Participants

This study was part of a larger investigation and details of the study participants are presented in Table 1 adapted from (Mcglory et al., 2019). Twenty healthy, young females (18–30 years) took part in this study. Participants were recreationally active, defined as performing structured exercise no more than twice weekly. Exclusion criteria included any diagnosis of thrombosis, tobacco usage, and injury to the lower extremities within 1 year of the study. Additionally, those supplementing with n‐3 fish oil within 6 months of the study were excluded, as were those with chronic usage of anti‐inflammatory drugs or analgesics and/or diagnosed with chronic or acute illnesses. Use of oral contraceptives did not exclude females from participating in the study; however, those using oral contraceptives were evenly distributed between the intervention and control groups (*n* = 5/group). The trial was approved by the Hamilton Integrated Research Ethics Board (HIREB‐1457) and adhered to the standards set by the Canadian Tri‐Council Policy on the ethical use of human subjects in research (Canadian Institutes of Health Research, Natural Sciences and Engineering Research Council of Canada, Social Sciences and Humanitites Research Council of Canada, 2014) and the Declaration of Helsinki. Participants provided written informed consent prior to their inclusion in the study. Diet and physical activity were controlled before immobilization as previously described (Mcglory et al., 2019). Participants started the immobilization phase of the trial during the follicular phase of their menstrual cycle.

**TABLE 1:** Participant characteristics.

	Omega‐3 FA (*n* = 11)	Control (*n* = 9)
Age (yr)	22 ± 3	22 ± 3
Height (cm)	165 ± 7	164 ± 5
Weight (kg)	63.7 ± 8.0	63.0 ± 6.8
Body Mass Index (kg/m^2^)	23.1 ± 2.1	23.9 ± 2.5
Body fat (%)	33 ± 6	29 ± 6

*Note*: Data shown are means ± SD.

Abbreviations: cm, centimeters; kg, kilograms; m, meters; Yr, years.

### 2.2 Study design

**FIGURE 1:**
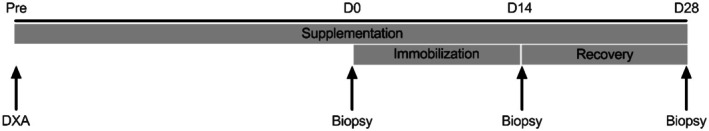
A schematic diagram of experimental design.

The full experimental design of this randomized, double‐blind, placebo‐controlled, repeated‐measures trial has been reported previously (Mcglory et al., 2019). Briefly, following consent and screening, participants reported to the laboratory on 4 occasions during an 8 week period to complete this study (Figure 1). At the baseline visit, participants were randomized via computer‐generated sequence to the n‐3 intervention (*n* = 11) or control (*n* = 9) groups as previously described (Mcglory et al., 2019). Supplements were administered as 20 mL doses of n‐3 (2.97 g EPA and 2.03 g DHA) or 20 mL doses of an isoenergetic sunflower oil (oleic acid content: 75%) for the intervention and control groups, respectively (Infinit Nutrition, Windsor, ON, Canada) and were consumed daily throughout the 8 week study. Both supplements were coconut flavor to mask the taste of the n‐3 supplement to maintain blinding. Following 4 week of supplementation, participants then underwent 2 week of single‐leg immobilization followed by 2 week recovery. Immobilization was accomplished using a Donjoy X‐ACT knee brace (Djo, Vista, CA, USA) with the leg fixed at a 60° angle of flexion between the thigh and shank, as previously described (Mcglory et al., 2019). After 2 weeks of immobilization, participants returned to remove the knee brace and were permitted to resume their preimmobilization activities, marking the start of the two‐week recovery phase. Biopsies were obtained from the *vastus lateralis* using a modified 5 mm Bergström needle adapted for manual suction using 1% xylocaine local anesthesia following an overnight fast prior to immobilization (D0), following immobilization (D14), and following recovery (D28). Muscle tissue was immediately stored in 2% glutaraldehyde for transmission electron microscopy (TEM) analysis or flash frozen in liquid nitrogen and stored at −80°C for Western blot analysis.

### 2.3 Mitochondrial content determination using transmission electron microscopy

Due to limited sample availability, determination of mitochondrial content using transmission electron microscopy (TEM) was only possible for 12 participants (n‐3: *n* = 7, control: *n* = 5). All sample preparation for transmission electron microscopy was completed by individuals at the McMaster University Medical Centre Electron Microscopy facility. Samples obtained from muscle biopsies were dehydrated in ethanol. Fixing was achieved with 1% osmium tetroxide, after which point samples were embedded in Spurr's resin. Longitudinal slices were sectioned at a thickness of 90 nm using a Leica UCT ultramicrotome, and set on copper grids. Samples were then stained with uranyl acetate and lead citrate for 5 and 3 min, respectively. Following staining, a thin carbon coating was added. Samples were then viewed and imaged at 5800x on a transmission electron microscope (Phillips CM10TEM, Japan). To ensure unbiased results, sixteen images per sample were taken in a random systematic order at each timepoint with 8 images taken of the intermyofibrillar (IMF) region and 8 images of the subsarcolemmal (SS) region by an individual blinded to condition. Of the images taken of the SS region, 6 included a nucleus and 2 did not. The IMF region was further divided into the peripheral IMF (within the first 3 sarcomeres from the SS region) and central IMF (remaining IMF region) regions as recent evidence suggests that the peripheral IMF region may represent the most metabolically active region of the muscle fiber as mitochondrial content is greatest in this region (Nielsen, Mogensen, et al., 2010) and small peripherally located IMCL are associated with higher whole body lipid oxidation (Covington et al., 2017). Images were analyzed using ImageJ software (Version 1.51a, National Institute of Health, USA). Mitochondria area density was determined as previously described (Tarnopolsky et al., 2007). We chose to report mitochondrial area density as a percentage to acknowledge the two‐dimensional nature of the images, which as a result does not accurately reflect the actual volumes of the structures but rather the percentage of the areas covered. As mitochondria‐intramyocellular lipid (IMCL) interactions are indicative of a greater capacity for fat oxidation (Kim et al., 2024; Miner et al., 2023), we also determined the percentage of mitochondria in apposition with IMCL.

### 2.4 SDS‐PAGE and Western blotting

Western blotting was performed to assess the content of proteins involved in autophagy, apoptosis, mitochondrial fission, and mitochondrial fusion. 25 mM Tris buffer [25 mM Tris, 0.5% (v/v) Triton X‐100, protease inhibitor tablet (11,836,170,001, Roche Diagnostics, Laval, QC, Canada), and a phosphatase inhibitor tablet (4,906,845,001, Roche Diagnostics, Laval, QC, Canada)] was prepared on ice and added to all samples, which were then homogenized using a bead homogenizer (Tissuelyser II, Qiagen, Hilden, Germany). Total muscle protein content was determined using the bicinchoninic acid assay (B9643‐1 L, Sigma Aldrich, Saint Louis, MO, USA). Working samples of equal concentration were prepared in Laemmli sample buffer (0.5 M Tris–HCl, glycerol, 10% SDS, 1% bromophenol blue, β‐mercaptoethanol, and ddH2O). 10 μg of protein per sample, a calibration curve, and a protein ladder (1,610,373, Precision Plus Protein Standard, Bio‐Rad, Hercules, CA, USA) were run on 4–15% Criterion TGX Stain‐Free protein gels (5,678,085, Bio‐Rad, Hercules, CA, USA) for 45 minutes at 200 V. Proteins were transferred to a PVDF membrane (1,620,177, Bio‐Rad, Hercules, CA, USA) using a TransBlot Turbo Transfer System (Bio‐Rad, Hercules, CA, USA). The Chemidoc MP imaging system (Bio‐Rad, Hercules, CA, USA) was used to image the proteins on the gel and PVDF membrane pre‐ and posttransfer to ensure proper transfer of the proteins and to determine total protein posttransfer. All membranes were incubated in a blocking solution (either 5% or 2.5% (w/v) dry skim milk in TBS‐Tween: 20 mM Tris base, 137 mM NaCl, and 0.1% (v/v) Tween 20, pH 7.5) for 2 h. After blocking, immunoprobing with the proteins' respective primary antibody solutions occurred overnight at 4°C. The primary antibodies are as follows: LC3B (1:1000, Cell Signaling Technology‐2775; Danvers, MA, USA), BNIP3 (1:500, Sigma Aldrich‐B7931; St. Louis, MO, USA), SQSTM1 (1:2000, MBL International‐PM045; Woburn, MA, USA), procaspase‐3 (1:1000, Sigma Aldrich‐C8487; St. Louis, MO, USA; 1:1000, CS‐9664S, Danvers, MA, USA), procaspase‐9 (1:1000, Santa Cruz Biotechnology‐56,076; Dallas, TX, USA), BAX (N‐20) (1:1000, Santa Cruz Biotechnology‐493; Dallas, TX, USA), BCL‐2 (C‐2) (1:1000, Santa Cruz Biotechnology‐7382; Dallas, TX, USA), FIS1(C‐9) (1:1000, Santa Cruz Biotechnology‐376,469; Dallas, TX, USA), OPA1 (D‐10) (1:100, Santa Cruz Biotechnology‐393,296; Dallas, TX, USA). Membranes were washed (3 × 5‐min) in TBS‐Tween (20 mM Tris base, 17 mM NaCl, and 0.1% (v/v) Tween 20, pH 7.5) and then incubated with the appropriate anti‐mouse (1,706,516, Bio‐Rad, Hercules, CA, USA) or anti‐rabbit (1,706,515, Bio‐Rad, Hercules, CA, USA) IgG conjugates with horseradish peroxidase conjugated secondary Ab for 1 h at room temperature. Membranes were washed again (3 × 5‐min) in TBS‐Tween (20 mM Tris base, 17 mM NaCl, and 0.1% (v/v) Tween 20, pH 7.5) and then incubated in Clarity (1,705,061, Bio‐Rad, Hercules, CA, USA) or Clarity Max (1,705,062, Bio‐Rad, Hercules, CA, USA) Western ECL Blotting substrates for subsequent protein band detection with chemiluminescent imaging on the Chemidoc MP imaging system (Bio‐Rad, Hercules, CA, USA). The band density was assessed using Image Lab software (Version 6.0.1, Bio‐Rad, Hercules, CA, USA), and protein content was normalized within and between blots using total protein and the standard curve obtained from the gel.

### 2.5 Statistical analysis

Statistical analyses were performed using R Statistical Software (version 4.0.4; The R Foundation for Statistical Computing, Vienna, Austria). The normality was assessed using the Shapiro–Wilk test. If it was determined that the dataset was not normally distributed, it was transformed and tested for normality again. Comparisons were made using a two‐way mixed model ANOVA, with supplementation (control, n‐3) serving as the between factor and time (D0, D14, D28) as the within factor. Significant interactions and main effects were decomposed with Tukey's posthoc analysis. Where a significant change in mitochondrial content was observed, correlational analyses were performed to examine if the extent of the change was related to mitochondrial content in that region at baseline. Statistical significance was set to *p <* 0.05 for all statistical analyses.

## 3 RESULTS

Effects of immobilization and recovery with and without n‐3 supplementation have been reported previously (Mcglory et al., 2019). Briefly, quadriceps muscle volume (cm^3^) decreased in both control and n‐3 supplemented groups, but the absolute decline was greater in controls (controls: −150 ± 20 cm^3^ (~14%) vs. n‐3: −77 ± 18 cm^3^ (~8%), *p* < 0.01) and recovery to baseline following 2 weeks of remobilization was achieved in the n‐3 group only (Mcglory et al., 2019). The same was observed for peak quadriceps CSA (mm^2^), whereby both groups declined with immobilization, but the absolute decline was greater in controls (controls: −914 ± 116 mm^2^ (~14%) vs. n‐3: −535 ± 109 mm^2^ (~8%), *p* < 0.01) with recovery to baseline in the n‐3 group only (Mcglory et al., 2019). Lastly, there was a significant decrease (~6%) in leg lean mass in the control group after immobilization, which was restored with recovery, while no change was observed in the n‐3 group between timepoints (Mcglory et al., 2019).

**FIGURE 2:**
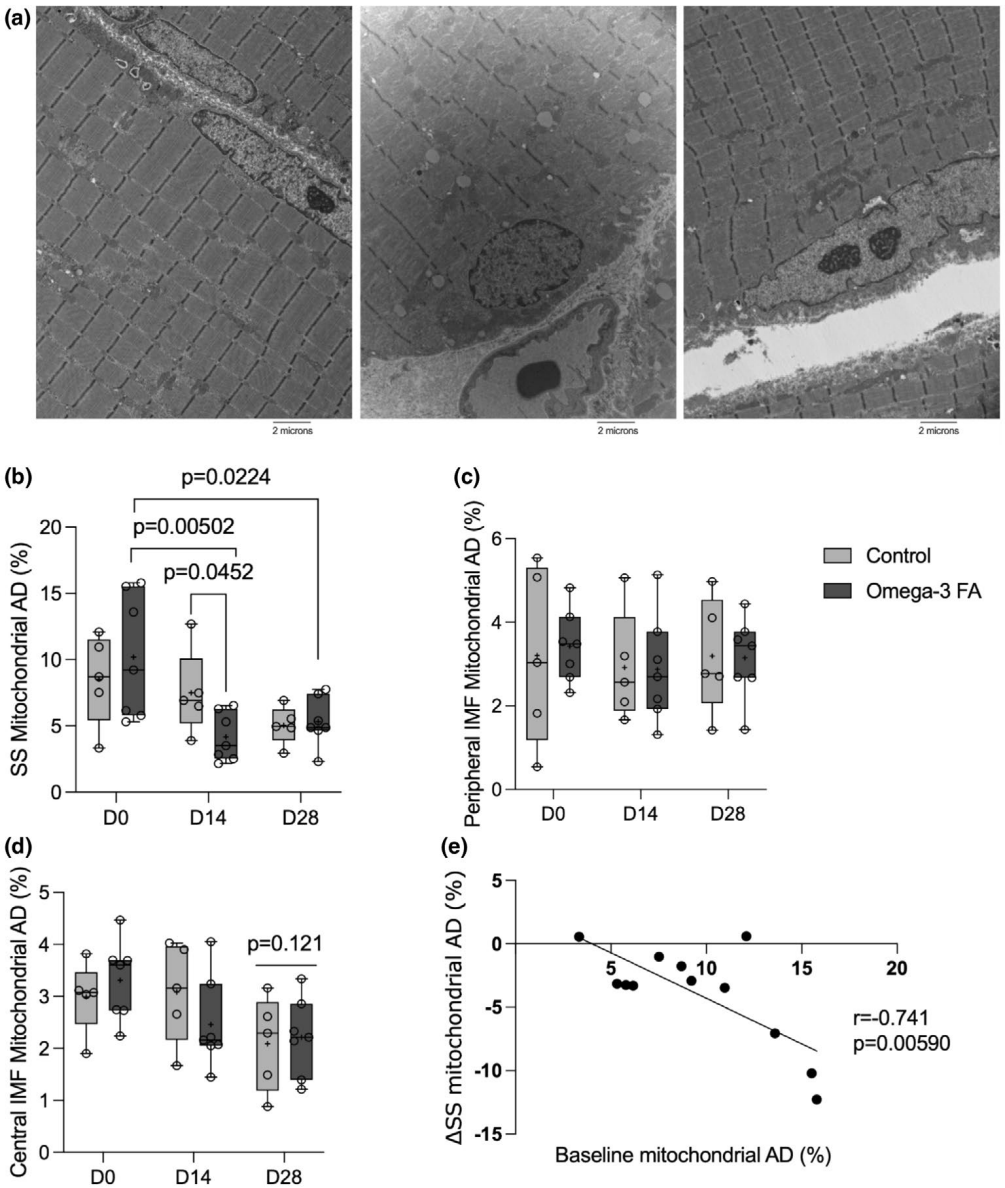
Effects of immobilization and recovery on mitochondrial content. (a) Representative images prior to immobilization (D0, left), after 2‐weeks of immobilization (D14, middle), and after 2 weeks of recovery (D28, right)from a control individual; (b–d) box and whisker plots showing mitochondrial area density (%) in controls (*n* = 5) and omega‐3 FA (*n* = 7) supplemented individuals prior to immobilization (D0), after 2‐weeks of immobilization (D14) and after 2 weeks of recovery (D28) in the (B) (SSC) peripheral IMF and (d) central IMF. Comparisons made with 2‐way mixed model ANOVA; (e) correlational analyses showing the negative relationship between baseline SS mitochondrial content and the change in SS mitochondrial content induced by disuse. For panels b–d the line within the confines of the boxplot represents the median and the cross represents the mean, while the whiskers and borders of the boxplot represent the extreme values and interquartile range (IQR), respectively. AD, area density; FA, fatty acid; IMF, intermyofibrillar; SS, subsarcolemmal.

Mitochondrial area density (%) in the SS, peripheral, and central IMF regions in the control and n‐3 supplemented groups are shown in Figure 2. Mitochondrial area density in the SS region declined during immobilization (*p* = 0.00933) and remained lower than baseline (D0) following recovery (*p* = 0.00452). There was no main effect of supplementation on mitochondrial area density in any region (SS *p* = 0.757, peripheral IMF *p* = 0.944, central IMF *p* = 0.890); however, the supplementation by time interaction indicated that SS mitochondrial area density was lower following immobilization (*p* = 0.00502) and recovery (*p* = 0.0224) compared with baseline in the n‐3 group, not the control group. Furthermore, SS mitochondrial area density was higher in the control group than the n‐3 group following immobilization (*p* = 0.0452). Correlational analyses indicated that there was a negative relationship between baseline SS mitochondrial content and the change in SS mitochondrial content during immobilization in both groups (*r* = −0.741, *p* = 0.00590). Mitochondrial content in the peripheral IMF region did not change during immobilization or recovery (*p* = 0.577). In the central region, mitochondrial area density was not different from baseline following immobilization (*p* = 0.369), but was lower than baseline following recovery (*p* = 0.0121). Similar results to central IMF were found when the effects of disuse and recovery on total IMF (central + peripheral) content were examined (*p* = 0.0204, data not shown). Correlational analyses indicated that there was no relationship between initial central IMF mitochondrial content and the change in mitochondrial content from baseline to postrecovery in both groups (*r* = 0.318, *p* = 0.314, data not shown). There was no effect of supplementation and no supplementation by time interaction for mitochondrial area density in the peripheral IMF or central IMF region.

**FIGURE 3:**
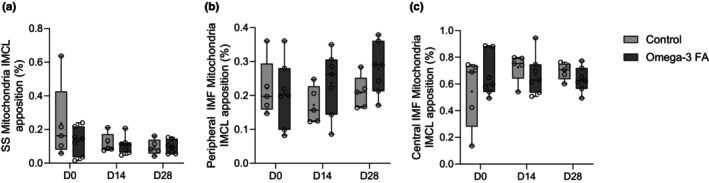
Box and whisker plots showing proportion of mitochondria (%) in contact with IMCL in the (a) SS, (b) peripheral IMF, and (c) central IMF regions prior to immobilization (D0), after 2 weeks of immobilization (D14) and after 2 weeks of recovery (D28) in controls (*n* = 5) and omega‐3 FA supplemented individuals (*n* = 7). Comparisons made with 2‐way mixed model ANOVA. Central IMF mitochondria‐IMCL apposition data (3C) was not able to be transformed for normal distribution. The line within the confines of the boxplot represents the median and the cross represents the mean, while the whiskers and borders of the boxplot represent the extreme values and interquartile range (IQR), respectively. AD, area density, FA, fatty acid, IMCL, intramyocellular lipid; IMF: Intermyofibrillar; SS, subsarcolemmal.

Physical contact interfaces between mitochondria and IMCL is thought to be an indicator of lipolytic capacity (Gemmink et al., 2017) and reflective of insulin sensitivity (Gemmink et al., 2017), and thus may reflect the overall health of the muscle cell. As such we wanted to examine whether this interaction decreased during disuse and whether omega‐3 supplementation influenced this effect. The proportion of mitochondria (%) in contact with IMCL in the SS, peripheral IMF and central IMF in the control and n‐3 supplemented groups are reported in Figure 3. n‐3 supplementation did not affect the proportion of mitochondria in contact with IMCL in any region (SS *p* = 0.388; peripheral IMF *p* = 0.085, central IMF *p* = 0.992). There was a large effect size (*ƞ* ^2^ = 0.144) indicating that the proportion of mitochondria in contact with IMCL in the SS region declined during immobilization and remained lower following recovery; however, this did not reach statistical significance (*p* = 0.055). There was no effect of immobilization or recovery on the proportion of mitochondria in contact with IMCL in the peripheral, or central regions (peripheral IMF *p* = 0.535, central IMF *p* = 0.228), and there was no supplementation by time interaction in any region (SS *p* = 0.318, peripheral IMF *p* = 0.441, central IMF *p* = 0.189).

**FIGURE 4:**
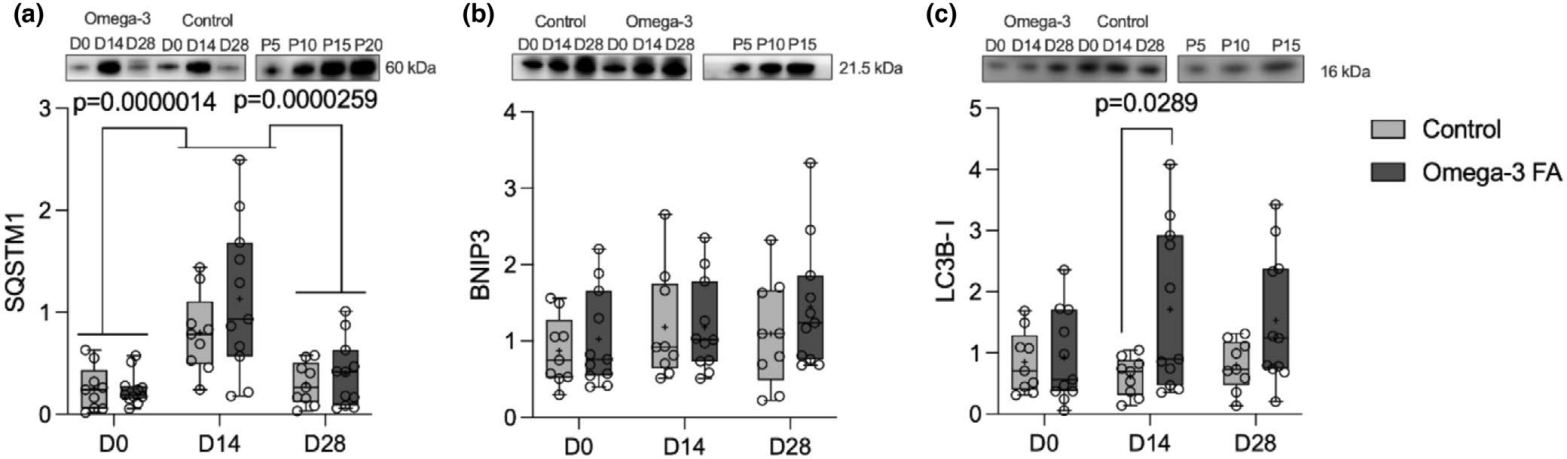
Representative blots and their accompanying box and whisker plots showing the content of autophagic proteins prior to immobilization (D0), after 2 weeks of immobilization (D14), and after 2 weeks of recovery (D28) in controls (*n* = 9) and omega‐3 FA supplemented individuals (*n* = 11). (a) SQSTM1; (b) BNIP3; and (c) LC3B‐I. Comparisons made with 2‐way mixed model ANOVA. The line within the confines of the boxplot represents the median, and the cross represents the mean, while the whiskers and borders of the boxplot represent the extreme values and interquartile range (IQR), respectively. Participants were staggered for supplementation, and all samples were run alongside a standard curve from a pool of all samples on each gel. D0, Day 0, prior to immobilization; D14, Day 14, post‐2 weeks of immobilization; D28, Day 28, post‐2 weeks of recovery; FA, Fatty acid; P5, 5 μg pooled sample, P10, 10 μg pooled sample, P20, 20 μg pooled sample. For full blots with marker ladder proteins, please see Figure S1A.

Disuse is associated with a pro‐inflammatory state and cellular stress (Slavin et al., 2024). Autophagy manages cellular stress by removing inflammasome complexes and damaged organelles, including mitochondria (Pang et al., 2022). Thus, we aimed to investigate whether the content of key autophagic proteins increased with disuse, and if omega‐3 supplementation impacted this effect. The content of autophagy proteins in the control and the omega‐3 FA supplemented groups is reported in Figure 4. SQSTM1 content was higher following immobilization compared with baseline (*p* = 0.0000014) and postrecovery (*p* = 0.0000259), with no difference between preimmobilization and recovery (*p* = 0.705). There was a main effect of time on BNIP3 content (*p* = 0.009); however, posthoc tests revealed no significant differences in BNIP3 content between any time points. LC3B‐I content did not change during immobilization or recovery (*p* = 0.092); however, there was a supplementation by time interaction indicating that LC3B‐I was greater in the n‐3 group compared with control following immobilization (*p* = 0.0289). LC3B, particularly LC3B‐II, is in low abundance in human skeletal muscle (Mizushima & Yoshimori, 2007; Rag et al., 2015). We could not consistently detect LC3B‐II in this cohort and thus could not quantify LC3B‐II in this analysis. There was no effect of n‐3 supplementation on the content of SQSTM1 (*p* = 0.265), BNIP3 (*p* = 0.549) or LC3B‐I (*p* = 0.085) and no further supplementation by time interactions (SQSTM1 *p* = 0.331, BNIP3 *p* = 0.236).

**FIGURE 5:**
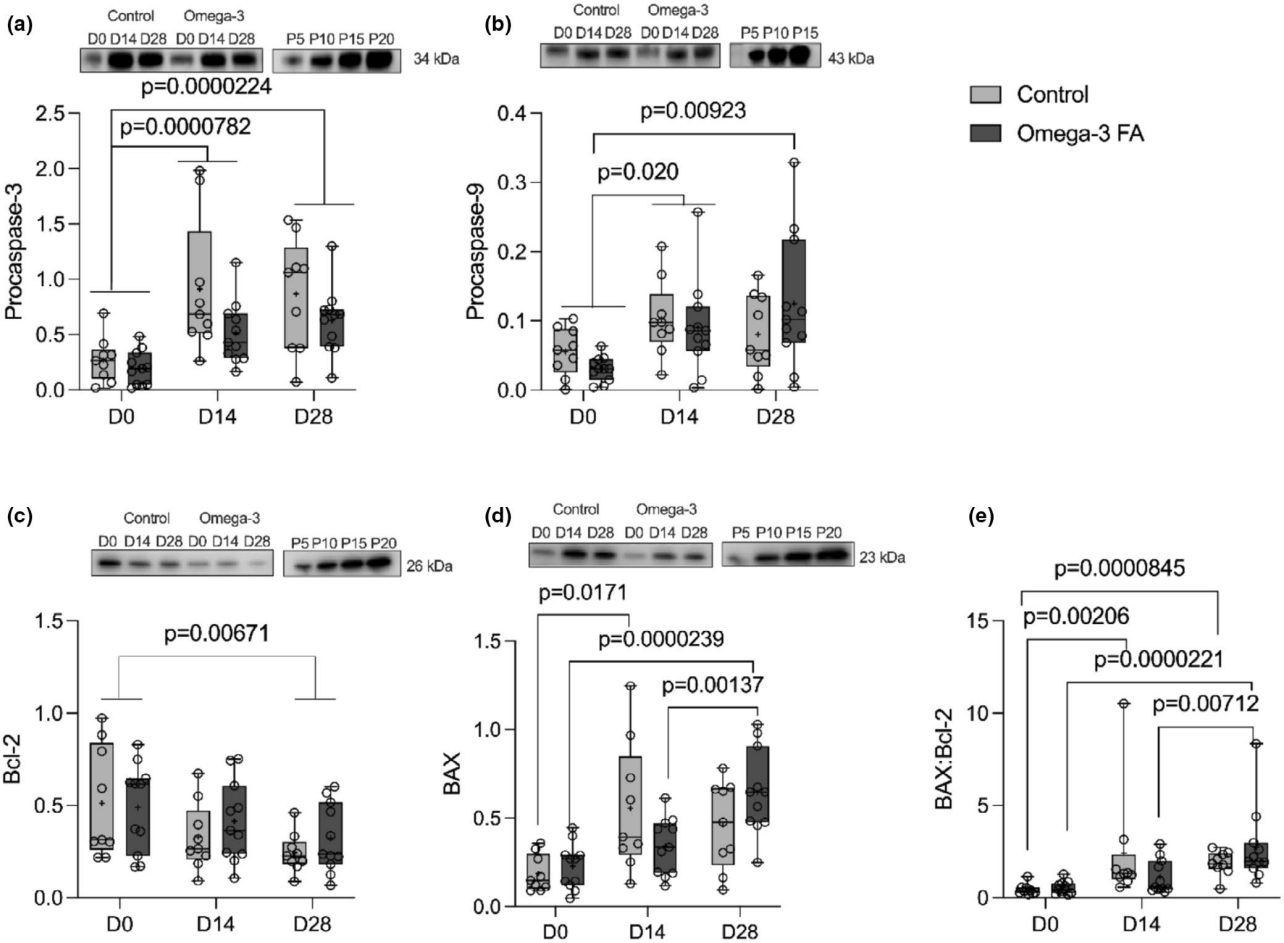
Representative blots and accompanying box and whisker plots showing content of intrinsic apoptotic proteins at baseline (D0), following 2‐weeks of immobilization (D14) and 2‐weeks of recovery (D28) in controls (*n* = 9) and omega‐3 FA supplemented individuals (*n* = 11). (a) procaspase‐3; (b) procaspase‐9; (c) Bcl‐2; (d) BAXand (e) BAX:Bcl‐2. Comparisons made with 2‐way mixed model ANOVA. Procaspase‐3 and BAX:Bcl‐2 data were transformed to achieve normality prior to ANOVA. The line inside the boxplot represents the median, and the cross represents the mean, while the whiskers and borders of the boxplot represent the extreme values and interquartile range (IQR), respectively. Participants were staggered for supplementation, and all samples were run alongside a standard curve from a pool of all samples on each gel. D0, Day 0, prior to immobilization; D14, Day 14, post‐2 weeks of immobilization; D28, Day 28, post‐2 weeks of recovery; FA, fatty acid; P5, 5 μg pooled sample; P10, 10 μg pooled sample; P20, 20 μg pooled sample. For full blots with marker ladder proteins, please see Figure S1B.

Disuse‐related atrophy is strongly associated with intrinsic apoptosis (Dupont‐Versteegden, 2006; Libera et al., 1999; Vazeille et al., 2008). A critical event in intrinsic apoptosis is mitochondrial release of cytochrome‐c into the cytosol, which in turn activates the caspase cascade and leads to cell death (McIlwain et al., 2015). Thus, we investigated the effects of immobilization on the content of key proteins in mitochondrial outer membrane permeabilization and the intrinsic apoptotic caspase cascade, and whether omega‐3 supplementation altered the response. The content of intrinsic apoptosis proteins in the control and omega‐3 FA supplemented groups are reported in Figure 5. There was no effect of supplementation on the content of any apoptosis proteins (*p* > 0.05). Both pro‐caspase 3 and 9 were higher than baseline following immobilization (pro‐caspase 3: *p* = 0.0000782 pro‐caspase 9: *p* = 0.020) and recovery (pro‐caspase 3: *p* = 0.0000224; pro‐caspase 9: *p* = 0.00593). There was no supplementation by time interaction for pro‐caspase 3 content (*p* = 0.565), but the supplementation by time interaction indicated that pro‐caspase 9 was only higher than baseline following recovery in the n‐3 group (*p* = 0.00923). Bcl‐2 was higher at baseline than after recovery (*p* = 0.00671) with no supplementation by time interaction (*p* = 0.273). BAX was higher than baseline after immobilization (*p* = 0.00796) and following recovery (*p* = 0.0000335), which was due to interactions by which BAX was greater than baseline after immobilization (*p* = 0.0171) but not recovery in the control group, while in the n‐3 supplemented group, BAX was greater after recovery compared with baseline (*p* = 0.0000239) and following immobilization (*p* = 0.00137). The BAX:Bcl2 ratio was higher than baseline following immobilization (*p* = 0.0000744) and continued to increase during recovery (*p* = 0.0244). The supplementation by time interaction indicated that BAX:Bcl2 increased during immobilization in the control group (*p* = 0.000206) and remained above baseline following recovery (*p* = 0.0000845). Alternatively, in the n‐3 group the BAX:Bcl2 ratio did not increase during immobilization, but was greater than baseline (*p* = 0.0000221) and postimmobilization (*p* = 0.00712) following recovery.

**FIGURE 6:**
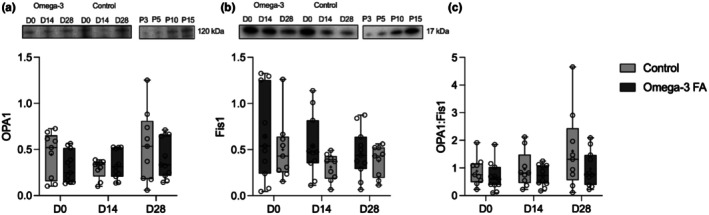
Representative blots and accompanying box and whisker plots showing protein content of mitochondrial fission and fusion proteins at baseline (D0), following 2‐weeks of immobilization (D14) and 2‐weeks of recovery (D28) in controls (*n* = 9) and omega‐3 FA supplemented individuals (*n* = 11). Comparisons made with 2‐way mixed model ANOVA. OPA1 data was not able to be transformed for normal distribution. (a) OPA1, (b) Fis1, and (c) ratio of OPA1 to Fis1. The line within the confines of the boxplot represents the median and the cross represents the mean, while the whiskers and borders of the boxplot represent the extreme values and interquartile range (IQR), respectively. Participants were staggered for supplementation, and all samples were run alongside a standard curve from a pool of all samples on each gel. D0, Day 0, prior to immobilization; D14, Day 14, post‐2 weeks of immobilization; D28, Day 28, post‐2 weeks of recovery; FA, fatty acid; P3, 3 μg pooled sample; P5, 5 μg pooled sample; P10, 10 μg pooled sample. For full blots with marker ladder proteins, please see Figure S1C.

Elevated fission and decreased fusion during disuse contributes to accumulated reactive oxygen species (ROS) damage of mitochondrial DNA, which can eventually lead to mitochondrial dysfunction, altered morphology, and losses in content (Iqbal et al., 2013; Iqbal & Hood, 2015; Westermann, 2008). As such, we aimed to investigate the effects of immobilization on the content of fission and fusion related proteins to determine whether the balance shifted toward fission with disuse, and if omega‐3 supplementation influenced this effect. Mitochondrial fission and fusion related protein content data are shown in Figure 6. There was a main effect of time for OPA1 (*p* = 0.017), Fis1 (*p* = 0.009) and the OPA1:Fis1 ratio (*p* = 0.033); however, posthoc analysis did not indicate any significant differences between timepoints. There was no effect of supplementation and no supplementation by time interaction for OPA‐1 (supplementation: *p* = 0.400, interaction: *p* = 0.173), Fis1 (supplementation: *p* = 0.244, interaction: *p* = 0.395) or OPA1:Fis1 ratio (supplementation: *p* = 0.147, interaction: *p* = 0.490).

## 4 DISCUSSION

We discovered that mitochondrial area density declined in the SS region (control: −12%, n‐3: −63%) of the muscle fiber after 2 weeks of immobilization and remained lower (control: −41%, n‐3: −42% vs. baseline) following 2 weeks of recovery. Contrary to our hypothesis, the decline in SS mitochondrial content was driven by a greater effect of immobilization in the n‐3 supplemented group; however, this may reflect the greater mitochondrial content in the n‐3 group at baseline. Interestingly, IMF mitochondrial content remained stable during immobilization, but central IMF mitochondrial content (control: −34%, n‐3: −32%) was lower than baseline following 2 weeks of recovery. In line with these findings, proapoptotic proteins pro‐caspase 3 and 9 and BAX increased during immobilization and remained elevated following recovery while antiapoptotic Bcl‐2 decreased only after recovery. SQSTM1, a marker of autophagy, increased during immobilization but returned to baseline after recovery. Concerning supplementation, LC3B‐I was greater in the n‐3 group, suggesting greater autophagy during immobilization with n‐3. Furthermore, n‐3 supplementation appears to delay apoptotic signaling as BAX content increased earlier in controls. The BAX:Bcl‐2 ratio, indicative of an apoptotic state, increased in controls during immobilization and remained elevated, whereas in the n‐3 group it rose only after recovery. This study's TEM‐based subcellular analysis provides novel insights into disuse‐induced mitochondrial remodeling, and inclusion of female participants addresses a critical knowledge gap in muscle physiology.

Our finding that 2‐weeks of limb immobilization reduced SS but not IMF mitochondrial content in young healthy females aligns with prior data in males showing a 20% decline in SS mitochondrial content after 2‐weeks of limb immobilization (Nielsen, Suetta, et al., 2010). Furthermore, our data are in line with exercise training trials that report a greater effect of training to increase SS mitochondrial content (Howald et al., 1985; Nielsen, Mogensen, et al., 2010) and trials in rats showing that respiration declined following 7 days of denervation (Slavin et al., 2024) and 2 days of hindlimb immobilization (Pang et al., 2022) in SS, but not IMF mitochondria. However, our findings differ from those of Ferreti and colleagues who reported a 17% decline in IMF mitochondria and a nonsignificant 11% reduction in SS mitochondria following 42‐days of bed rest (Ferretti et al., 1997). Based on these findings, we suggest that there may be a temporal effect of muscle disuse on mitochondria whereby SS mitochondria are immediately impacted at the onset of disuse, whereas IMF mitochondria are not affected until later during disuse. In support of this hypothesis, work in rats found that respiration in SS mitochondria was 40% lower 7 days after denervation and remained lower at 21 days, whereas respiration in IMF mitochondria was not different from baseline at 7 and 14 days post denervation, but was 20% lower than baseline at 21 days post denervation (Slavin et al., 2024). Together, these findings suggest that at the onset of disuse SS mitochondria are particularly impacted; however, as the effects of disuse continue, IMF mitochondria are also impacted. Our findings confirm that in response to short‐term immobilization the decline in mitochondrial content is driven by effects in the SS region and support the hypothesis that SS mitochondria are more responsive to alterations in physical activity; however, future work should consider examining the time‐course of mitochondrial changes during disuse and how these changes impact the health of the cell.

To our knowledge, we are the first to describe subcellular localized effects of recovery from immobilization on mitochondria. Perhaps not surprising given that previous work has reported incomplete recovery of mitochondrial‐related genes following 2‐weeks of step reduction (McGlory et al., 2018) SS mitochondrial content remained ~42% below baseline following 2‐weeks of recovery in both groups. Despite no change in IMF mitochondrial content during immobilization, central IMF mitochondrial content declined ~33% postrecovery. These findings indicate that disuse effects on mitochondrial content persist into recovery and are not prevented by omega‐3 FA supplementation, emphasizing the need for robust rehabilitation practices to restore mitochondrial health and muscle function.

We also found that 2‐weeks of immobilization increased markers of autophagy and apoptosis. Autophagy markers returned to baseline in recovery, but apoptosis markers remained elevated and the antiapoptotic protein Bcl‐2 declined after recovery, further increasing the BAX:Bcl‐2 ratio. Although caspases are well‐known as mediators of apoptotic cell death, they are also vital in skeletal muscle differentiation and myogenesis (Fernando et al., 2002). For example, Caspase 3‐null myoblasts show impaired differentiation, and knockdown of Caspase 9 blunts myoblast fusion (Murray et al., 2008). In vivo inhibition of Caspase 3 promotes satellite cell self‐renewal but impairs muscle regeneration following injury due to the requirement for Caspase 3‐mediated PAX7 cleavage for satellite cell activation and differentiation (Dick et al., 2015). Thus, the degree of caspase activation appears critical in the skeletal muscle *death‐differentiation* response with high levels promoting death while low levels facilitate differentiation (Rahman & Quadrilatero, 2023a). This thesis is supported by evidence showing that restoring Caspase 3 or Caspase 9 to normal levels both limits myoblast apoptotic cell death and enhances myoblast differentiation (Baechler et al., 2019; McMillan & Quadrilatero, 2014; Wang et al., 2011). We also found elevated SQSTM1 during immobilization, consistent with prior rat work showing a 2.5 fold increase following immobilization, and that chronic inhibition of autophagy worsened immobilization‐induced skeletal muscle atrophy (Foresto et al., 2016). Autophagy is likewise required for proper skeletal muscle differentiation and myogenesis (Baechler et al., 2019; McMillan & Quadrilatero, 2014), together suggesting elevated Caspase levels along with elevated autophagic signaling likely reflect coordinated remodeling during immobilization and recovery.

Contrary to our hypothesis, n‐3 FA supplementation did not attenuate mitochondrial content loss. Rather, SS mitochondrial content decreased to a greater extent in the n‐3 FA group during immobilization, which likely reflects either the greater baseline mitochondrial content in our n‐3 group (34% higher) rather than a stronger immobilization effect or enhanced clearance of damaged/dysfunctional mitochondria through autophagic/mitophagic signaling. Although BNIP3 did not differ between groups, alternative mechanisms, such as PINK‐PRKN‐mediated mitophagy may be involved (Rahman & Quadrilatero, 2023b). LC3B‐I was higher in the n‐3 group, suggesting differential autophagy signaling that warrants further investigation to determine its impact on mitochondrial content and function in immobilized muscle.

The greater SS mitochondrial content decline with n‐3 supplementation during disuse appears inconsistent with previous data from this cohort showing preserved submaximal ADP‐stimulated mitochondrial respiration and oxidative phosphorylation (OXPHOS) protein content in the n‐3 group (Miotto et al., 2019). IMF mitochondria are reported to have a greater abundance of electron transport chain proteins and contribute more to energy production (Ferreira et al., 2010) whereas SS mitochondria primarily mediate signal transduction and substrate transport (Ritov et al., 2005). Considering that IMF mitochondria represent a greater proportion of total mitochondrial content and did not change during disuse, our findings complement rather than contradict other findings. Moreover, our findings suggest that the disuse‐related declines in OXPHOS content and mitochondrial respiration in controls likely reflect IMF mitochondrial dysfunction, rather than mitochondrial content loss. In line with this, BAX and the BAX:Bcl‐2 ratio rose only in controls after immobilization, suggesting greater mitochondrial dysfunction leading to increased apoptotic signaling. Given that mitochondrial cristae density displays plasticity in response to exercise training (Nielsen et al., 2017; Schytz et al., 2024), future trials could examine whether disuse reduces mitochondrial cristae density and how this relates to OXPHOS protein content and mitochondrial respiration.

As with any trial, this study has its limitations. Findings are limited by the small sample size available for mitochondrial content measurements by TEM, particularly for the control group, which may have reduced power to detect subtle differences. Furthermore, Western blot analyses were performed on whole muscle homogenates, so signaling could not be evaluated relative to subcellular localization. As a result, we cannot determine whether disuse influenced the content of autophagy, apoptosis, and mitochondrial fission and fusion proteins in specific mitochondrial subpopulations. Furthermore, we were unable to assess autophagic flux (e.g., marker accumulation in the presence of inhibitors) which would provide a more robust understanding of how disuse, recovery, and n‐3 supplementation influence autophagy. Unfortunately, we were limited by available samples; the majority had been sectioned for TEM analyses and other cohort investigations, with remaining samples in archived homogenates for WB purposes and not viable tissue. Future work should isolate SS and IMF fractions to link region‐specific protein content with corresponding structural and functional changes as well as assess mitophagic flux.

In conclusion, 2‐weeks of unilateral limb immobilization reduced SS mitochondrial content, with a greater decline in the n‐3 group. SS mitochondrial content remained lower and central IMF mitochondrial content declined during recovery, independent of supplementation. Autophagy and apoptosis markers increased during immobilization, with autophagy markers returning to baseline during recovery, while apoptosis markers did not. Our findings indicate that immobilization and remobilization exert phase‐specific effects: SS mitochondrial content declines in immobilization, while IMF mitochondria are affected after recovery. Lastly, the preservational effects of omega‐3 FA supplementation on mitochondrial function and muscle mass during disuse appear independent of changes in mitochondrial content.

## AUTHOR CONTRIBUTIONS

C.M., S.M.P., and M.D.A. designed the research. C.M., M.K., and R.B. conducted the research and performed sample collection. M.M.L. and M.N.B. performed sample analyses and analyzed the data. M.M.L. prepared the figures and drafted the manuscript that was edited by M.C.D. All authors edited and approved the final version of the manuscript. M.M.L. assumes final responsibility for the integrity of the data. The authors declare no conflicts of interest.

## FUNDING INFORMATION

This work was supported by a Natural Sciences and Engineering Research Council of Canada (NSERC) Discovery Grant to M.D.A, NSERC Canada Graduate Scholarships (MSc) to M.M.L and M.N.B, Research Fellowships from Diabetes Canada and the European Society for Clinical Nutrition and Metabolism to C.M. and a Canadian Institutes of Health Research Award to S.M.P.

## Supporting information


Data S1.

## Data Availability

Data are available from the corresponding author upon request.
