# Supplementary material for: Omega‐3 fatty acid supplementation does not attenuate declines in skeletal muscle mitochondrial area in young, healthy females during immobilization
Source: Physiol Rep. 2026 Jan 23;14(2):e70736. doi: 10.14814/phy2.70736 (PMC12830871; doi:10.14814/phy2.70736)
Supplement: Supplementary file 1 — Data S1. [file PHY2-14-e70736-s001.docx]

**Omega-3 fatty acid supplementation does not attenuate declines in skeletal muscle mitochondrial area in young, healthy females during immobilization: Supplementary Data**


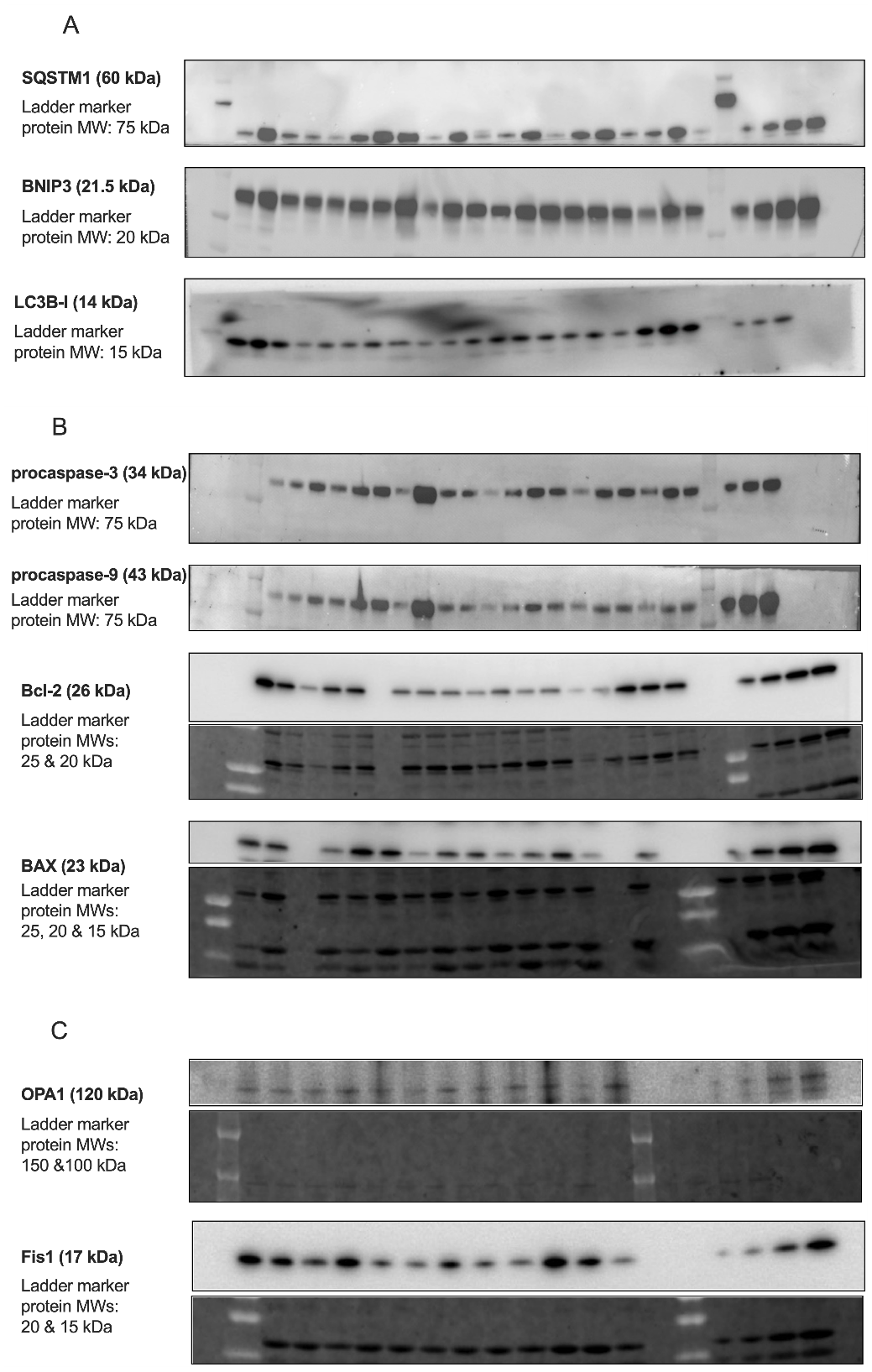


**Supplementary Figure 1:** Sample blots with labelling of the nearest marker ladder molecular protein weight for A) Autophagic proteins: SQSTM1, BNIP3 and LC3B-I; B) Intrinsic apoptosis proteins: procaspase-3, procaspase-9, Bcl-2, and BAX; and C) mitochondrial fission and fusion proteins OPA1 and Fis1. For Bcl-2, BAX, OPA1 and Fis1, overlay of colorimetric and chemiluminescent images was not possible; they are accompanied by stain free images of their respective blots with negatives of the reference ladder proteins.


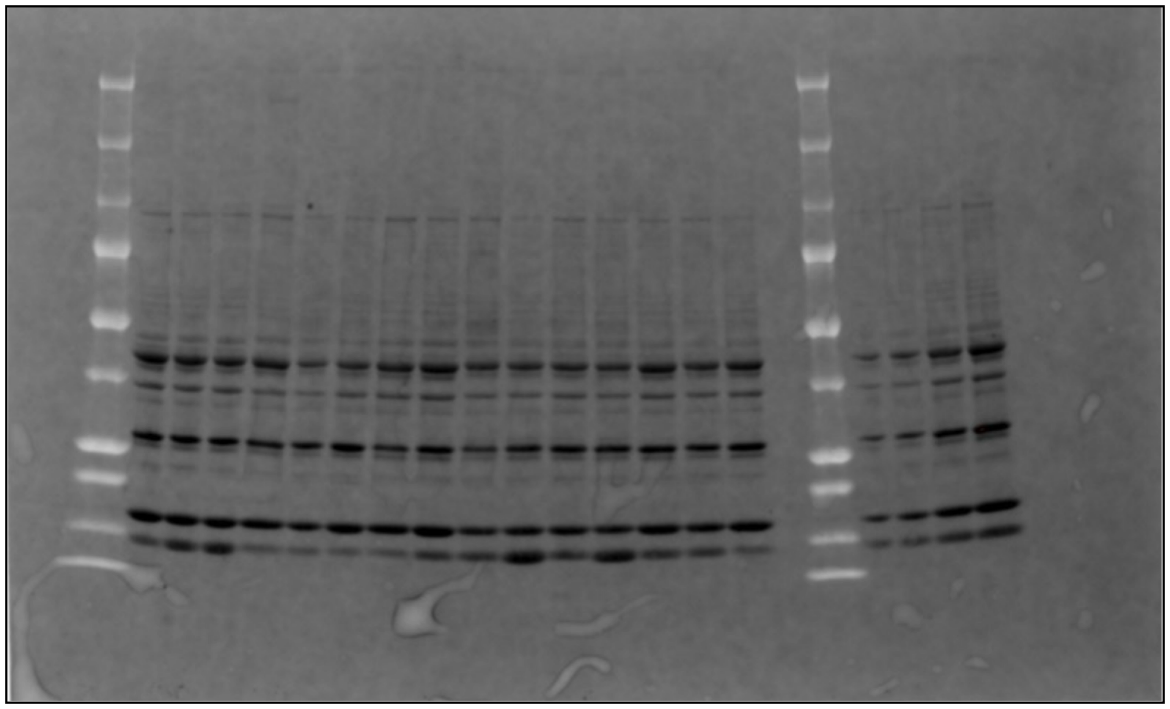


**Supplementary Figure 2**: A representative image of the post-transfer stain free blot to show total protein content to show equal loading.
